# CRISPR Variants for Gene Editing in Plants: Biosafety Risks and Future Directions

**DOI:** 10.3390/ijms242216241

**Published:** 2023-11-13

**Authors:** Ali Movahedi, Soheila Aghaei-Dargiri, Hongyan Li, Qiang Zhuge, Weibo Sun

**Affiliations:** 1 Department of Biology and the Environment, College of Life Sciences, Nanjing Forestry University, Nanjing 210037, China; ali_movahedi@njfu.edu.cn (A.M.); lhy1110@njfu.edu.cn (H.L.); qzhuge@njfu.edu.cn (Q.Z.); 2 Department of Biological Control Research, Iranian Research Institute of Plant Protection, Agricultural Research Education and Extension Organization (AREEO), Tehran 19858-13111, Iran; s.aghaei6418@gmail.com

**Keywords:** CRISPR variants, gene editing, plant genetic improvement, biosafety, off-target effects

## Abstract

The CRISPR genome editing technology is a crucial tool for enabling revolutionary advancements in plant genetic improvement. This review shows the latest developments in CRISPR/Cas9 genome editing system variants, discussing their benefits and limitations for plant improvement. While this technology presents immense opportunities for plant breeding, it also raises serious biosafety concerns that require careful consideration, including potential off-target effects and the unintended transfer of modified genes to other organisms. This paper highlights strategies to mitigate biosafety risks and explores innovative plant gene editing detection methods. Our review investigates the international biosafety guidelines for gene-edited crops, analyzing their broad implications for agricultural and biotechnology research and advancement. We hope to provide illuminating and refined perspectives for industry practitioners and policymakers by evaluating CRISPR genome enhancement in plants.

## 1. Introduction

Using CRISPR-based methods for modifying plant genomes has led to significant advancements in enhancing plant genetics. Researchers have discovered different CRIS-PRs, including Cas9, Cpf1, and Cas12a, which can improve specific plant genes [1]. With the advancement of precise gene editing techniques, it is now feasible to develop plants with favorable traits, such as increased productivity, enhanced disease resistance, and improved nutritional composition [2].

While CRISPR-based gene editing is a powerful tool for improving plants, it raises concerns and challenges related to biosafety [3]. One major challenge is off-target effects, where unintended mutations occur in non-targeted genome regions [4]. This issue can be reduced by using advanced CRISPR variants like Cas12b (C2c1), which offer higher specificity and accuracy [5]. Another concern is the potential for unintended outcomes, including allergens or pollutants. Therefore, strict biosafety assessments are necessary to ensure the safety of genetically modified plants for human consumption and the environment [6]. Several regulatory frameworks have been developed to address the biosafety issues associated with gene-edited plants. The United States Department of Agriculture (USDA) has stated that gene-edited plants will not be regulated if they are produced through conventional breeding methods (Secretary Perdue Issues USDA Statement on Plant Breeding Innovation|USDA. https://www.usda.gov/media/press-releases/2018/03/28/secretary-perdue-issues-usda-statement-plant-breeding-innovation (accessed on 19 April 2023)). In the European Union (EU), gene-edited plants have the same regulations as genetically modified organisms (GMOs) (GMO legislation. (n.d.). Food Safety. https://food.ec.europa.eu/plants/genetically-modified-organisms/gmo-legislation_en (accessed on 19 April 2023)). Addressing the related biosafety concerns is as important as harnessing the benefits of CRISPR-based gene editing in plants. A comprehensive understanding of its molecular characteristics is essential before a genetically modified plant can be considered for market release. This involves analyzing the specific genomic modifications introduced through CRISPR and genome editing techniques. Modern molecular biology tools such as next-generation sequencing and bioinformatic analysis detect and characterize these modifications, ensuring the accuracy and predictability of the genetic changes made [7]. A safety assessment regarding human health is also crucial. This includes evaluating toxicology, allergenicity, and nutritional value. Toxicological studies assess whether consuming the modified plant poses any adverse effects on human health. Allergenicity assessments aim to identify potential allergenic proteins introduced during genetic modification. Nutritional value analysis ensures that the modified plant maintains or improves its essential nutrients, promoting its potential benefits for human nutrition [8]. Assessing the environmental impact of genetically modified plants is equally important. Factors such as gene flow to wild or non-target plants, potential ecological effects on non-target organisms, and implications for beneficial insects are evaluated. Understanding the potential impact on the ecosystem helps in designing risk management strategies and mitigates their intended environmental consequences [9]. Furthermore, post-market environmental monitoring becomes essential once genetically modified plants are released. This involves systematic and ongoing surveillance of the modified plants in the environment. This monitoring aims to detect any unexpected effects, assess the long-term stability and behavior of the modified plants in the ecosystem, and validate the accuracy of the predictions made during pre-release risk assessments. Guided by scientific hypotheses, monitoring these efforts proactively identifies and manages potential environmental concerns [10]. This comprehensive review explores the potential of CRISPR-based genome editing technology in plants and its different aspects. It discusses the effectiveness of Cas9, Cpf1, and Cas12a variants for precise gene editing in plants. This paper also highlights the importance of considering biosafety concerns, such as minimizing unintended effects and ensuring risk assessments are conducted for plants used in cultivation, food, and feed. It explores the role of nanotechnology in further enhancing biosafety measures for CRISPR genome editing. This review discusses international guidelines for biosafety in gene-edited crops and addresses specific concerns regarding potential allergens and toxins. Finally, it offers insights into future directions for improving plant biosafety using CRISPR-based gene editing.

## 2. Conventional Mutagenesis in Plant Genome Modification

Conventional mutagenesis methods, including chemical, physical, and biological mutagenesis, have played a significant role in plant breeding and genetic research. These methods offer a means to introduce random genetic variations that can lead to desirable plant traits [11]. However, it is essential to note that conventional mutagenesis generates a wide range of mutations throughout the genome [12]. With advancements in genomics and molecular techniques, combining traditional mutagenesis with precise genetic engineering tools holds successful promise for accelerating crop improvement efforts in the future. Here, two commonly used methods of conventional chemical and physical mutagenesis are presented as follows.

### 2.1. Chemical Mutagenesis

Chemical mutagenesis involves treating plants with chemical compounds that can induce DNA damage and mutations [13]. Ethyl methanesulfonate (EMS) is a chemical often used to generate plant mutations. It replaces a hydrogen atom in the DNA with an ethyl group, causing changes in the DNA sequence, such as base substitutions, deletions, and insertions. The effectiveness of EMS in inducing mutations depends on its concentration, and it is crucial to find the right balance between generating enough mutations and avoiding harm to the plants [14,15].

### 2.2. Physical Mutagenesis

Physical mutagenesis involves subjecting plants to various forms of radiation or high-energy particles, which cause DNA damage and induce mutations [16]. Gamma rays and X-rays are commonly used sources of radiation. These forms of radiation induce structural changes in the DNA, such as double-strand breaks and chromosomal rearrangements in cells [17]. Ultraviolet (UV) radiation, specifically UV-B and UV-C, is also used to induce mutations [18]. Physical mutagenesis has been used in crops to develop improved varieties with beneficial traits, including breeding [19,20].

## 3. The Promise and Potential of Primarily CRISPR-Based Genome Editing Technology in Plants

CRISPR-based technology has been used to enhance the characteristics of different plants, offering a promising approach to improving plant genetics. An exciting application of this is in the enhancement of disease resistance in crops. *MLO* loci have been targeted by RNA-guided Cas9 endonucleases in three plant species: bread wheat, tomato, and grapevine, which were reported to be susceptibility genes, with homozygous loss-of-function mutants increasing the resistance to powdery mildew of barley, Arabidopsis, and tomato plants [21,22,23]. Bread wheat plants mutated by CRISPR/Cas9 in one (*TaMLO-A1*) of the three *MLO* homeo-alleles have showed improved resistance to *Blumeria graminis* f. sp. tritici infection [21]. In tomatoes, *SlMLO1* was targeted at two sites and self-pollinated to generate CRISPR/Cas cassette-free individuals, resulting in a non-transgenic variety of tomato fully resistant to *Oidium neolycopersici* [23]. In grapevines, the molecular feasibility of a *VvMLO7* knockout has been demonstrated through CRISPR/Cas9 RNP in protoplasts, but no plants were regenerated [22]. Rice has been developed to resist bacterial blight disease caused by the γ-proteobacterium *Xanthomonas oryzae* pv. *oryzae* through CRISPR/Cas9 mutagenesis in *OsSWEET13* [24].

Another study focused on cassava, a vital food crop in Sub-Saharan Africa. This study targeted a gene involved in plant immunity that increased resistance to cassava mosaic virus [25]. Strategies primarily based on CRISPR have exhibited the capability to improve the dietary quality of vegetation. Utilizing the CRISPR/Cas9 generation, researchers genetically modified corn plants to increase the biosynthesis of beta-carotene, a key component for vitamin A. According to Li et al. [1], this experimental corn vegetation exhibited a significant increase in beta-carotene levels, measuring 169 times higher than the control variety. This finding shows that using CRISPR-based genome enhancement may additionally function in a viable manner to cope with vitamin A deficiencies in underdeveloped areas worldwide. Another study used CRISPR/Cas9 to regulate the genes of potato tubers to lower levels of acrylamide, a carcinogen that forms when potatoes are fried at excessive temperatures [2]. The new CRISPR/Cas9 generation has changed farming by making crops more resistant to environmental degradation. For example, a CRISPR/Cas9-APOBEC1 base editing system targeted the rice *NRT1.1B* gene to replace C/T in the target site, Thr327Met, to improve the efficiency of its nitrogen use [26]. This breakthrough has significantly increased grain productivity under nitrogen-deprived conditions. Therefore, CRISPR-mediated techniques hold immense potential for shaping the future of sustainable agriculture [27]. The potential of CRISPR-based genome editing technology in plant genetic improvement extends beyond crop traits. Researchers have shown the possibility of using CRISPR/Cas9 to edit the genes responsible for flower color in morning glory plants. The edited plants showed a range of new colors, showcasing the versatility of CRISPR-based techniques in developing plants with novel traits [28]. These studies show the enormous potential of CRISPR-based genome editing technology for increasing crop productivity, nutritional costs, and sustainability. Addressing the challenges and biosafety concerns associated with CRISPR-based total gene enhancement is critical for ensuring the safety of genetically modified plant life for human consumption and the environment.

## 4. The Precision and Efficiency of Cas9, Cpf1, and Cas12a Variants for Targeted Gene Editing in Plants

The CRISPR–Cas system is an exact and efficient tool for targeted gene editing in plants. This system comprises RNA-guided Cas enzymes, including Cas9, Cpf1, and Cas12a, which recognize specific target sequences within the plant genome and introduce site-specific DNA breaks [29]. Natural repair mechanisms within the plant cell can then repair these breaks, leading to precise, targeted changes in the gene sequences. One of the key advantages of the CRISPR–Cas system for gene editing is its excessive precision. Studies have proven that Cas9 and Cpf1 have extreme specificity in targeting particular plant sequences with minimum off-target consequences [30]. This is a critical advantage for avoiding unintended genetic changes, maintaining the integrity of the plant genome, and increasing the accuracy of gene editing.

**Figure 1:**
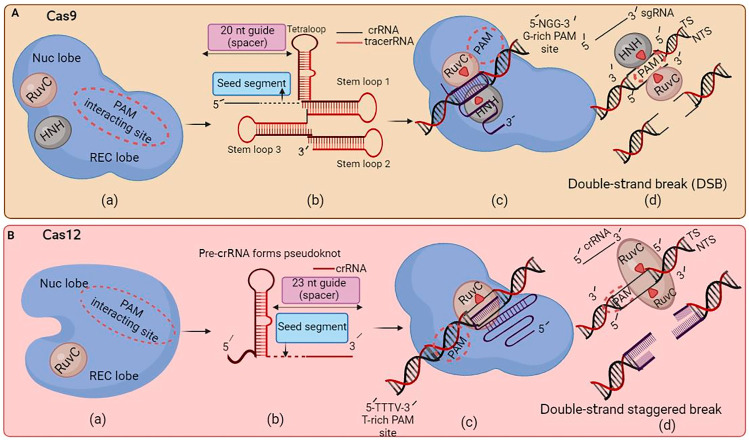
The critical differences between Cas9 and Cas12a are illustrated here. (**A**): (**a**) Cas9 has two endonuclease domains that, when activated, use the RuvC and HNH domains to target the target strand (TS) and non-target strand (NTS). (**b**) Cas9 needs tracrRNA for the synthesis of mature crRNA. (**c**) For the cleavage of the target site, the PAM of Cas9 requires NGG-rich areas. (**d**) Cas9 produces blunt ends while concurrently breaking both the TS and NTS. (**B**): (**a**) Cas12a uses RuvC, a single endonuclease domain, to cut the TS and NTS. (**b**) Cas12a generates mature crRNA without the assistance of tracrRNA. (**c**) The Cas12a PAM requirements are “TTN/TTTN”, with a preference for AT-rich areas. (**d**) Cas12a cleaves the NTS first, then the TS, resulting in a double-strand staggered break (sticky ends).

Moreover, the efficiency of CRISPR–Cas gene enhancement in flora has constantly increased with the improvement of high-fidelity variations. For instance, a study by Zhong et al. [31] showed that using a Cas9 variant with better specificity led to higher editing performance in rice vegetation with a substantially decreased rate of off-target mutations. Cas9 is a multidomain protein with two lobes: the nuclease (NUC) and recognition (REC) lobes. The NUC consists of endonuclease domains HNH and RuvC and a protospacer adjacent motif (PAM)-interacting domain, targeting both target strands (TSs) and non-target strands (NTSs). The REC lobe has multiple α-helical recognition domains that bind to RNA and DNA, ensuring targeted DNA sequences. Cas9 has the potential for uses in medical research and studying genetic changes in disease susceptibility and treatment. However, ethical concerns arise, particularly when affecting human embryos (Figure 1(Aa)). The tetraloop and stem-loop stabilize the gRNA structure, while the CRISPR RNAs (crRNAs) guide Cas9 to the target DNA sequence. The *trans*-activating crRNA (tracrRNA) helps process the crRNA and aids its complex formation with Cas9. On the other hand, the seed segment is responsible for base pairing with the target DNA sequence, ensuring binding specificity (Figure 1(Ab)). CRISPR–Cas9 advances genetic engineering, enabling precise genome editing in organisms, particularly those with G-rich PAM sites, but concerns about potential off-target effects and unintended consequences persist (Figure 1(Ac)). Cas9 breaks the TS and NTS using HNH and RuvC domains, causing blunt ends. The double-strand break (DSB) can be repaired using non-homologous DNA end joining (NHEJ) or homology-directed repair (HDR), which can introduce insertions or deletions, leading to gene knockout. NHEJ is error-prone and requires a donor template, while HDR introduces precise changes, like point mutations or exogenous DNA insertions (Figure 1(Ad)). Cas12a is a CRISPR-associated protein with a unique mechanism that uses RuvC to cut TSs and NTSs, providing higher specificity and reducing off-target effects. Its single active site for DNA cleavage and strand displacement makes it an attractive genome editing tool (Figure 1(Ba)). Cas12a has emerged as a promising alternative to Cas9 and Cpf1 for plant-targeted gene editing. This enzyme is smaller than Cas9 and Cpf1 and recognizes different target sequences, providing greater flexibility in target selection [32]. Cas12a generates mature crRNA without tracrRNA assistance, forming pseudoknots with a 23 nt guide spacer. This efficient targeting simplifies the process and reduces the off-target effects (Figure 1(Bb)). Figure 1(Bc) illustrates the Cas12a PAM requirements, with particular attention to AT-rich areas and a specific sequence of “TTN/TTTN”. Cas12a cleaves the NTS first, followed by the TS, causing a double-strand staggered break (Figure 1(Bd)). Cas12a is smaller than Cas9, making it easier to deliver into cells. Cas9 has been broadly used for genome improvement packages, while Cas12a has garnered interest for its capability to target and cleave RNA molecules, providing a potential tool for growing diagnostic exams. The CRISPR–Cas system has constantly been evolving and improving, considering its initial improvements, and Cas12a has emerged as a promising alternative to Cas9 and Cpf1 for targeted gene editing in plants [33]. CRISPR–Cas tools show high precision and performance in plants, enabling crop improvement and breeding programs. By targeting specific genes or traits, researchers can enhance plant resistance to pests, improve their dietary content, and increase their sensitivity to environmental stresses [33].

## 5. The Biosafety Concerns Regarding Genetic Manipulation Using CRISPR Variants, Enhancing Specificity, and Minimizing Off-Target Effects

The CRISPR–Cas system has modified genetic engineering by providing a specific and efficient tool for manipulating gene sequences in multiple organisms, representing a significant milestone in scientific advancement. However, tits unparalleled potential for off-target outcomes engenders significant concern among biosafety experts, as inadvertent genomic loci may be cleaved and modified, leading to harmful consequences such as altered gene expression, disrupted cellular processes, and unintended genetic mutations that might cause pathological conditions. Mitigating the prospect of off-target effects is imperative for responsible research and development in this field. Researchers have been investigating advanced CRISPR variants, such as Cas12b, that offer superior specificity and efficacy in DNA targeting, minimizing the likelihood of off-target cleavage and attenuating biosafety concerns. Cas12b is significantly smaller than Cas9 or Cas12a, making it easier to deliver into cells. A recent study suggested that Cas12b could be reprogrammed to target particular sequences with high specificity and efficiency in various plant cells [34]. Cas12c, also known as C2c3, is a CRISPR variant that shares structural similarities with Cas12a. Cas12c has been used to edit the genomes of bacteria and mammalian cells with high precision and flexibility [29]. The small size of Cas12c might also facilitate its delivery into cells. However, it is also susceptible to off-target effects, which pose significant biosafety concerns [35]. These off-target effects often occur because of the sequence homology of the target loci [36]. Hence, choosing unique target sequences in the genome can be a strategic approach to reducing off-target effects [37]. The detection and evaluation of these off-target effects are crucial for ensuring biosafety. Several genome-wide methods have been developed for this purpose, such as integration-deficient lentiviral vectors (IDLVs) [38]; chromatin immunoprecipitation sequencing (ChIP-seq) [39]; breaks labeling, enrichment on streptavidin and next-generation sequencing (BLESS) [40]; genome-wide unbiased identification of DSBs enabled by sequencing (GUIDE-seq) [41]; differential cellular indexing of transcriptomes via sequencing (DISCOVER-seq) [42]; and genome-wide off-target analysis via two-cell embryo injection (GOTI) [43]. However, each method has limitations; for instance, IDVLs have a limited off-target detection efficiency of 1%, and BLESS requires a reference genome [44]. Moreover, deep sequencing is used to measure off-target mutations that may occur at frequencies ranging from 0.01% to 0.1% [45], which highlights the importance of a robust pipeline design for data analysis to provide crucial insights into the off-target effects of CRISPR systems, including Cas12c [44]. Therefore, while Cas12c has unique advantages, careful strategies are required for its delivery into cells to ensure biosafety and address the potential off-target effects with long-term safety implications. Cas13 is an additional CRISPR variant with properties that allow its application in RNA editing and detection. Cas13a is being explored for its potential to treat or prevent diseases caused by RNA viruses, such as SARS-CoV-2 [46]. The Cas13a-based diagnostic test enables rapid, low-cost virus detection, with a 100 copies/μL sensitivity under 30 min [46]. However, the use of such biotechnological tools brings about biosafety concerns. The manipulation of genetic material, especially of a potentially harmful virus like SARS-CoV-2, requires stringent safety measures to prevent accidental release or misuse. The Cas13a-based test involves recognizing and ligating viral RNA, which entails handling viral genetic material [47]. Therefore, robust biosafety protocols are essential to protect researchers and the environment from potential exposure to the virus. Additionally, as Cas13a has been proposed as a preventative or therapeutic tool against RNA viruses [48], careful clinical testing and regulation are required to ensure its safe and effective use in humans. Biosafety is a pivotal aspect of CRISPR–Cas technologies, largely because of the potential for off-target effects that could inadvertently damage healthy cells or cause unintended genetic alterations. Therefore, enhancing the specificity of Cas enzymes is a key focus in ongoing research. Chen et al. [49] found that using Sniper-Cas9, a modification of the original SpCas9, substantially decreased off-target effects. Their study showed a 10-to-100-fold reduction in off-target cleavage, validating the engineered enzyme’s improved fidelity. Importantly, on-target efficiency was largely unaffected, with the Sniper-Cas9 performing comparably to the wild-type SpCas9. Similarly, evoCas9 was developed to enhance target discrimination. Lee et al. [50] reported that this enzyme, created via directed evolution, reduced off-target effects by over 100-fold in certain situations. This significant reduction was achieved without compromising the on-target activity, highlighting the success of this engineered enzyme. However, it is important to remember that while these developments have vastly reduced off-target mutations, they have not solved them entirely. For instance, Tsai et al. [51] found that even with high-fidelity Cas9 variants, specific cell types still exhibited off-target mutations. This underlines the necessity for ongoing research to further enhance the specificity and safety of CRISPR–Cas technologies.

## 6. Risk Assessments for Plants in Cultivation, Food, and Feed Applications

Risk assessments play a crucial role in ensuring the safety and sustainability of plants developed for cultivation, food, and feed [52]. These assessments address the specific purpose of the plants and evaluate their potential impact on the environment, human health, and animal health. When evaluating plants intended for cultivation, the assessments focus on their potential invasiveness and impact on native plant species. Factors such as their reproductive capacity, ability to spread, and competition with local plants are carefully examined to prevent any harmful ecological consequences of their introduction into the environment [53,54]. For plants intended for food production, thorough safety assessments are conducted to ensure their suitability for human consumption. These assessments investigate the presence of allergens, toxins, or other harmful substances that could pose risks to human health [55]. Their nutritional composition and its potential effects on human well-being are also evaluated to guarantee safe and nutritious food derived from transgenic plants [55]. Plants developed for feed undergo assessments focusing on their safety and suitability for animal consumption. The nutritional value of these plants as animal feed has also been carefully evaluated to ensure they provide adequate nutrition and contribute positively to the well-being of the animals that consume them [56].

## 7. The Promising Role of Nanotechnology in Enhancing the Biosafety of CRISPR Genome Editing

CRISPR/Cas9 technology has revolutionized the field of genome editing, offering unparalleled precision and efficiency in gene manipulation. However, ensuring biosafety remains a paramount concern in its application. Recent advances in nanotechnology have presented a promising avenue in plants toward enhancing the biosafety of CRISPR/Cas9 technology, creating a potential combination for sustainable genome engineering [57,58]. Nanotechnology can significantly improve the delivery methods of CRISPR/Cas9 components by manipulating matter at the atomic and molecular scale [57]. Encapsulating the CRISPR/Cas9 system within nanoparticles can allow it to be delivered directly to the target cells, reducing off-target effects and enhancing precision [59,60]. Integrating nanotechnology and CRISPR/Cas9 technology is not confined to human health and medicine. For instance, sustainable agriculture can significantly benefit from this combination. Using nano-vectors for gene delivery in plant genome engineering promises more efficient and targeted genetic modifications, which could lead to crops with improved traits and higher disease resistance [61]. Importantly, the alliance between nanotechnology and CRISPR/Cas9 aligns with the National Academies of Sciences, Engineering, and Medicine (NASEM) recommendations. In their 2016 report on genetically engineered (GE) crops, NASEM highlighted the need for continued research and development to resolve the confusion surrounding GE crops [62]. By facilitating more precise and efficient genetic modifications, the nanotechnology–CRISPR/Cas9 combination could help to address these concerns, aligning with the broader goal of biosafety in genome editing [62]. In conclusion, integrating nanotechnology and CRISPR/Cas9 technology holds immense potential for enhancing the biosafety of genome editing.

## 8. International Biosafety Guidelines for Gene-Edited Crops

CRISPR, a powerful tool for gene editing, has greatly influenced agricultural and biotech research, especially in plant development. Furthermore, global biosafety guidelines also apply to gene-edited crops, impacting farming practices and biotechnical research and development. The Cartagena Protocol on Biosafety, adopted by different countries, regulates the safe transfer, handling, and use of living modified organisms (LMOs), which include gene-edited crops [63], while some countries have excluded specific applications of gene editing from their GMO regulations [63]. However, the European Food Safety Authority (EFSA) has affirmed that plants developed through novel genomic techniques, such as genome editing, are subject to environmental risk assessments (ERAs) before their release or placement on the market [64]. The European Court of Justice’s 2018 decision clarified that plants developed through directed mutagenesis are regulated as GMOs in the EU, and that the current regulatory framework for biotechnology products applies to organisms developed through novel genomic techniques [64]. Thus, some discourse is necessary to confirm the responsible use and regulation of gene-edited crops and to encourage sustainable agriculture [65]. The role of organizations such as the National Advisory Commission on Agricultural Biotechnology, the Food and Agriculture Organization (FAO), and non-governmental organizations (NGOs) in monitoring and managing gene-edited crops, which should be implemented regularly to detect unintended effects or environmental impacts when forming and establishing biosafety guidelines, becomes increasingly apparent.

## 9. Addressing Biosafety Concerns in Gene-Edited Plants: From Novel Allergens to Toxins

Improving gene-edited plants can revolutionize agriculture, improve crop yields, and augment meal production in diverse ways. However, there are issues regarding the biosafety of these plants, mainly concerning the capability to produce accidental outcomes, comprising the emergence of novel allergens or toxins that would affect human health and the environment. Gene editing techniques such as CRISPR/Cas9 provide potent tools for modifying the genetic editing of plants. However, there is a risk that this precision could create new genes or regulatory elements that could trigger biosafety issues [66]. For example, a recent study reported the creation of a new starch in a gene-edited potato that led to the production of acrylamide. This neurotoxin forms when certain foods are cooked at high temperatures [67]. Similarly, scientists have expressed concerns that gene editing could lead to novel plant allergens and unexpected toxicological effects [68]. To address these concerns, researchers have been exploring various strategies to reduce the biosafety risks associated with gene-edited plants. One approach uses bioinformatic tools to identify and characterize potential unintended effects before any environmental release (Table 1) [69]. For example, computational methods can screen the edited genomes for new or truncated genes and identify regions of homology with known allergens or toxins. Such pre-release testing can help to identify and mitigate the potential risks associated with off-target effects or unintended consequences. Another approach would be the use of effective risk assessment protocols to evaluate the safety of gene-edited plants. This method entails surveying the genetic modifications, followed by tests designed to assess the possibility of the emergence of allergens, toxins, or other biosafety issues (Table 1) [70]. Toxicological assays, feeding trials, and sensory evaluations are some examples of risk assessments. Such testing can aid in identifying any potential problems before the plants are released into the environment. Gene-edited plants have great potential to enhance agricultural productivity and food security. Still, their biosafety should be evaluated to minimize the potential risks of unintended effects, such as allergies and toxins. Comprehensive risk assessments and bioinformatic tools can help identify and mitigate the potential risks associated with gene editing. Regulatory frameworks incorporating these measures can help ensure the safe use of gene-edited crops in agricultural production.

## 10. Future Directions for CRISPR-Based Gene Editing in Plant Biosafety

Plant genome editing raises methodological, biosafety, and social concerns regarding target gene selection, RNA design, and delivery methods. One problem in genetic engineering is the possibility of producing accidental genetic alterations because of off-target mutations (Table 2) [71]. During the DNA repair method, it has been observed that fragments of CRISPR/Cas9 can undergo degradation and eventually become inserted into predicted or unexpected genomic locations [72]. The capacity for transgene integration and the possibility of off-target mutations can be avoided by managing pre-developed CRISPR/Cas9 ribonucleoproteins in vitro with diverse research [22,71]. While this approach has confirmed efficacy in many crop species, specific barriers remain that hinder its implementation: inadequate balance, multiplied charges, and worrying technical stipulations that cause refinement (Table 2) [22,73]. To reduce Cas9 off-target outcomes, efforts have been made to enhance RNA guide-designed techniques, ribonucleoprotein transport, protein engineering, spatiotemporal control of CRISPR/Cas9 and/or gRNAs, and synthetic genetic circuits to modulate CRISPR characteristics [71]. Base editing is being modified to enhance the specificity of base editors via extracting deaminase out of Cas9 binding using exclusively deaminase effectors or rationally engineering the deaminase in order to lower its DNA-binding potential [30]. The Cas9 protein can cause an immune response when delivered via a virus in plant cells (Table 2) [74]. The specificity of Cas9 and the limited availability of suitable target sites due to the PAM requirements can pose challenges and concerns in gene editing [75]. Protein engineering techniques have been employed to identify mutations in Cas9 proteins that can enhance its PAM efficiency, improve fidelity, and enable its recognition of alternative DNA motifs (Table 2) [76]. In plant cells, making specific improvements to the design of the Cas9 and guide RNA, such as by using FokI fusions, paired nicking, and truncated guide RNAs, can help enhance the specificity of gene editing [77]. It is extremely important to differentiate between genetically changed plants, transgenic flora, and genome-edited plants because the latter may also or may not fall under the class of transgenic plants. Disseminating knowledge on the essential standards of genome editing throughout the populace can rectify and avert the dissemination of erroneous beliefs (Table 2) [78,79]. However, this technology raises moral concerns regarding the long-term outcomes of ecosystems. CRISPR-based gene enhancement has great potential for improving agriculture; however, we must balance biosafety concerns with scientific advancements. Future directions should encompass growing plants with green photosynthesis, disorder resistance, and enhanced dietary exceptionality while ensuring the responsible use of gene-enhancing technology. As we move forward, we must consider the biosafety-related implications of these advances and ensure that CRISPR-based gene enhancement is used sustainably and responsibly in agriculture.

## 11. Conclusions

In recent years, scientists have been using a powerful technology known as CRISPR for editing plant genomes. This technology has sufficient possibilities to improve plants and solve worldwide agricultural problems. With CRISPR, scientists can target and edit specific plant genes, enhancing their growth, disease resistance, and nutritional value. It is a precise tool for modifying plant traits. However, biosafety concerns have emerged alongside these remarkable advancements, causing the need for a comprehensive risk assessment framework for CRISPR-edited plants. One major challenge is the potential for off-target effects, where unintended mutations occur in non-targeted regions of the genome. To address this, advanced CRISPR variants with higher specificity and accuracy, like Cas12b (C2c1), have been developed and can minimize off-target effects. To ensure the safety of genetically modified plants for human consumption and the environment, rigorous biosafety assessments must be conducted. This involves a multi-faceted approach, beginning with the molecular characterization of the genome-edited plants. Using the latest molecular biology tools, scientists can accurately identify and characterize the precise genomic modifications introduced through CRISPR editing techniques. This step ensures the predictability and accuracy of the genetic changes. The safety assessment for human health focuses on toxicology, allergenicity, and nutritional value. Toxicological studies evaluate whether consuming modified plants harms human health, while allergenicity assessments aim to identify potential allergenic proteins introduced during the genetic modification process. Additionally, nutritional value analysis ensures that the modified plants maintain or improve their essential nutrients, enhancing their potential benefits for human nutrition. Moreover, considering the environmental impact is equally critical. The assessment includes evaluating factors like possible ecological effects on non-target organisms and impacts on beneficial insects. Understanding these potential effects on the ecosystem is crucial for designing appropriate risk management strategies and mitigating any unintended environmental consequences. To further enhance biosafety measures, post-market environmental monitoring is necessary. This ongoing surveillance of genetically modified plants released into the environment helps detect any unanticipated effects, assesses their long-term stability and behavior in the ecosystem, and validates the accuracy of any predictions made during pre-release risk assessments. Expanding and refining the risk assessment protocols for CRISPR-edited plants is recommended. This includes continually improving the specificity and accuracy of CRISPR variants and applying emerging technologies like nanotechnology to enhance biosafety measures. International collaboration is crucial for establishing unified biosafety guidelines for gene-edited crops, achieving a balance between innovative technological advancements and rigorous safety considerations.

## Figures and Tables

**Table 1:** Strategies for minimizing biosafety risks of gene-edited plants.

Strategy	Description References	References
Pre-Release Testing	Uses bioinformatic tools to identify and characterize potential unintended effects.	[69]
Risk Assessment Protocols	Involves surveying the genetic modifications and tests for allergens and toxins.	[70]
Toxicological Assays	Comprises techniques for identifying toxic substances in edited plants.	[70]
Feeding Trials	Assesses the effects of consuming the edited plants on animals.	[70]
Sensory Evaluations	Tests for taste, smell, or texture changes in the edited plants.	[70]

**Table 2:** Challenges in plant genome editing and their potential solutions.

Challenges in Plant Genome Editing	Potential Solutions	References
Off-target mutations	Enhanced RNA guide-designed techniques, protein engineering, managing pre-developed CRISPR/Cas9 ribonucleoproteins in vitro	[22,71]
Inadequate stability, high costs, and technical requirements	Refinement of the genome editing process	[22,73]
Immunogenic response to the Cas9 protein,	Design improvements for Cas9 delivery methods, such as adeno-associated virus	[74]
Cas9 specificity, and limited site recognition due to PAM requirements	Cas9 and guide RNA, using Cas9 variants and other proteins like Cpf1 nucleases	[76,77]
Dissemination of erroneous beliefs	Dissemination of knowledge about genome editing among the public	[78,79]
